# Mesoporous Drug Delivery System: From Physical Properties of Drug in Solid State to Controlled Release

**DOI:** 10.3390/molecules28083406

**Published:** 2023-04-12

**Authors:** Yanan Wang, Fang Li, Junbo Xin, Jia Xu, Guanghua Yu, Qin Shi

**Affiliations:** 1 School of Pharmacy, Jiangsu Vocational College of Medicine, Yancheng 224005, China; 2 School of Pharmacy, Faculty of Health and Medical Science, Taylor’s University, Subang Jaya 47500, Malaysia

**Keywords:** mesoporous drug delivery system, polymorph control, confined crystallization, in vitro and in vivo performance, controlled release

## Abstract

Mesoporous materials, which exhibit great potential in the control of polymorphs and delivery of poorly water-soluble drugs, have obtained considerable attention in the field of pharmaceutical science. The physical properties and release behaviors of amorphous or crystalline drugs may be affected by formulating them into mesoporous drug delivery systems. In the past few decades, an increasing amount of papers have been written about mesoporous drug delivery systems, which play a crucial role in improving the properties of drugs. Herein, mesoporous drug delivery systems are comprehensively reviewed in terms of their physicochemical characteristics, control of polymorphic forms, physical stability, in vitro performance, and in vivo performance. Moreover, the challenges and strategies of developing robust mesoporous drug delivery systems are also discussed.

## 1. Introduction

Oral delivery of drugs with poor water solubility is one of the greatest challenges in the pharmaceutical field, which has aroused considerable interest in the past several decades [1]. An increasing number of new chemical entities exhibit higher lipophilicity, lower water solubility, and higher molecular weight, leading to lower oral absorption, and thus, to poor bioavailability. In order to improve their bioavailability, several strategies have been proposed, including particle size reduction, salt formation, cocrystal, coamorphous formulation, amorphous solid dispersions, cyclodextrin inclusion, and micro-emulsion [1]. 

One of the most promising approaches is the use of mesoporous materials to deliver poorly water-soluble drugs, which has been increasingly accepted and recognized for their potential use as dissolution enhancers [2,3,4,5]. Mesoporous silica, considered a “generally regarded as safe (GRAS)” material, is proposed to be an ideal matrix for optimizing drug loading and subsequent release [6,7,8]. The unique advantages of mesoporous materials determine their broad application prospects [9]. Mesoporous silica has high specific surface area and pore volume, which facilitates the construction of multiple surface active sites and renders it an attractive substrate for enhancing the dissolution of poorly water-soluble drugs. For instance, some ordered mesoporous silica materials such as MCM-41 and SBA-15 possess very large specific surface areas with a range of 500–1000 m^2^/g. These properties of high surface area and large pore volume render the mesoporous silica attractive substrates for enhancing the dissolution of poorly water-soluble drugs. Pore size and structure are also demonstrated to be the key factors affecting the role of mesoporous materials as a dissolution enhancer. From the perspective of pore structure, mesoporous materials used for different pharmaceuticals can be mainly divided into channel-like, cage-like, and continuous 3D pore form [2]. In addition, drug molecules loaded in the pores and on the silica surface of mesoporous materials are preferably in amorphous form [5].

Another hot topic of mesoporous materials in pharmaceutical field is the formation of nano-structures of amorphous drugs and their crystallization in a confined state [10,11]. The nanostructure, molecular dynamics, and thermodynamics of amorphous drugs are extensively investigated to reveal their crucial role in affecting physical stability and subsequent dissolution behaviors [10]. The drug phases under confinement in the mesoporous materials can also make them the nanoscale crystallization chambers for polymorph screening [12,13]. Considerable numbers of new polymorphs have been discovered for confined crystallization. Additionally, confined crystallization would also lead to the stabilization of some metastable crystal forms. 

In this review, we will focus on recent developments in mesoporous drug delivery systems from the perspective of polymorph control, physical stability, in vitro performance, and in vivo performance. In the first part, the function of mesoporous materials in the polymorph screening and molecular dynamics under confinement will be systemically overviewed, while in the second part, the recent advances in the studies related to the in vitro and in vivo performance of these mesoporous drug delivery systems will be introduced. Moreover, the role of mesoporous materials will be discussed in depth from the perspective of dimensional and interfacial effects as well.

## 2. Nano-Confinement Effect of Mesoporous Materials on the Molecular Dynamics of Amorphous Drug and Crystallization

### 2.1. Nano-Confinement Effect on Molecular Dynamics

The stabilization of amorphous drugs and polymorph screening have been research hotspots in the field of pharmaceutical science in the past decades [14,15,16,17,18]. Amorphization is one of the most widely used pharmaceutical approaches to improve the solubility and dissolution of poorly water-soluble drugs. However, amorphous solids tend to crystallize and lose their advantages in in vitro performance because of their higher energy [17,19]. It has been widely reported that the use of mesoporous materials provides promising results for maintaining the physical stability of amorphous solids [10,20,21,22]. The amorphous form of several drugs, including ibuprofen, griseofulvin, indomethacin, simvastatin, nifedipine, ezetimibe, and celecoxib, can be physically stabilized by using mesoporous materials [10,20,21,22]. Interestingly, a recent study revealed that mangiferin, a promising therapeutic polyphenol, can be transformed into its amorphous form by balling milling with mesoporous silica [23]. 

Exploring and understanding molecular dynamics is one of the key issues for the stabilization of amorphous drug in mesoporous systems. Knapik et al. investigated the effects of nano-confinement on the molecular dynamics and physical stability of amorphous ezetimibe in the mesoporous materials Aeroperl 300 and Neusilin US2 [21]. It was found that the crystallization of ezetimibe can be effectively inhibited by incorporating it into the pores of Neusilin US2, with an average pore diameter of ~5 nm. Whereas the amorphous ezetimibe confined in the pores of Aeroperl 300 with a larger pore diameter of ~30 nm tends to recrystallize. The molecular dynamics analysis of confined ezetimibe in these mesoporous systems reveals the existence of two distinguishable phases. One is attributed to the surface–liquid interface, while the other is connected with the core molecules in the inner pore volume. In addition, the estimated critical diameter of ezetimibe’s nuclei is also demonstrated to be slightly larger than the pores of Neusilin US2. Therefore, the significant stabilization of ezetimibe in Neusilin US2 is concluded as an interplay of the following three factors: the change of molecular mobility under nano-confinement, the immobilization effect of the pore wall, and a pore size smaller than the critical nuclei size. 

Similar stabilization effects have also been reported for menthol incorporated into mesoporous silica matrix with pore size of 3.2 and 5.9 nm, which has been evidenced by the observation of glass transition in thermal analysis [24]. Two relaxation processes of drug molecules can be identified in the pores of these silica matrices by dielectric spectroscopy. One faster model is proposed to be attributed to the mobility of neat-like menthol molecules (α-process), while the slower model is originated from the hindered mobility of menthol molecules adsorbed on the inner walls of the pore (S-process). The S-process is more intensive than α-process for both pore sizes. In the case of larger pore size (5.9 nm), the fraction of molecular population governing the α-process is greater, which also seems to be strongly related to the faster drug release at initial stage than in the 3.2 nm composite. In addition, the crystallization of menthol in the pore size of 3.2 nm can be effectively inhibited, which can also be attributed to the smaller dimensions compared to the critical size for nucleation.

The theoretical critical pore diameter, attracting considerable interest for amorphous solids, represents the maximum value below which spatial confinement can effectively inhibit the undesired crystallization of drugs. Vraníková et al. investigated the critical pore diameters of three drugs with poor glass-forming ability by using different mesoporous carriers, and monitored the physical stability of these mesoporous systems for over 3 months [25]. The three drugs exhibit different estimated critical pore diameters. It was also found that temperature dependence is barely related to storage condition. Among these drugs, benzamide exhibits the predicted relatively large critical pore diameter of 29.5 nm and superior physical stability. On the contrary, haloperidol exhibits impaired physical stability depending on drug loading, and has a much smaller critical pore diameter of 8.4 nm.

Recent studies also reveal that interfacial effect is one of the important factors influencing the molecular dynamics of confined drugs [10]. Moreover, if the long-range effect of interfacial interactions is considered, the formation of hydrogen bonding interaction should also be highlighted [10]. Zhang et al. investigated the molecular dynamics of indomethacin and griseofulvin confined in anodic aluminum oxide (AAO) template by dielectric and thermal analysis [10]. The crystallization of drugs confined in the pores of AAO and their *T*_g_s show evident pore-size dependence. The temperature dependence of α-relaxation exhibits the significant change upon cooling, which is mainly attributed to the vitrification of molecules at the interface and the local density heterogeneity under the isochoric confinement. A typical two-layer model can be used to describe the molecular dynamics of indomethacin confined in AAO template. For comparisons, griseofulvin confined in AAO template exhibit three *T*_g_s under fast cooling. This phenomenon of three *T*_g_s indicates the existence of a thermodynamic nonequilibrium interlayer between the interfacial layer and the bulk core. 

**Figure 1:**
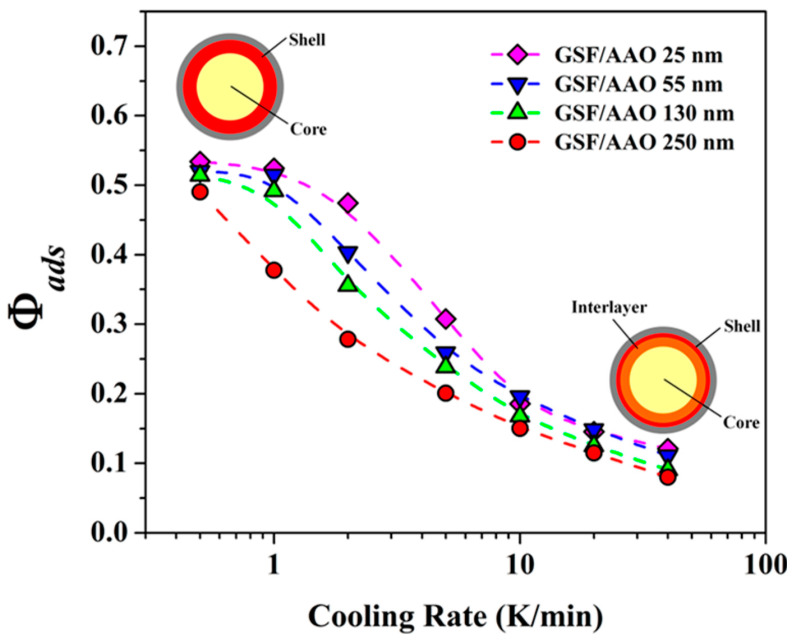
Percent of the change in heat capacity (*Φ*_ads_) for the *T*_g_ of the interfacial layer, as a function of cooling rate for griseofulvin confined in AAO templates with different pore diameters. *Φ*_ads_ is deduced from the change in heat capacity in the *T*_g,high_ region, can be calculated as △C_p,high_/(△C_p,high_ + △C_p,inter_ + △C_p,low_). For these cooling rate-dependency experiment, these confine samples are firstly heated above melting point and followed by a cooling process with different rates. *T*_g_ and ΔCp values are obtained from the subsequent heating curves with a 10 K/min rate. Adapted from the Ref. [10] with the permission. (Copyright © 2023 American Chemical Society).

Thermal analysis demonstrates that the content of interfacial layer represented by the change in heat capacity gradually reduces with the decrease of cooling rates (Figure 1). Even at high temperature, the very thin layer of molecules adjacent to the pore walls can still be clearly detected, which is mainly due to the restriction of the rigid surface. Furthermore, the evolution of the interfacial layer is also strongly correlated with the pore size of AAO. More rapid evolution can be observed with the decrease of pore size. These results are mainly attributed to the enhancement of the interfacial effect induced by the increase of the geometrical confinement effect, facilitating the accelerated stabilization of the interfacial region. By using the method of slow cooling (0.5 °C/min), the metastable “three-layer” model can be transformed into a stable core-shell nanostructure. Additionally, through the surface modification of AAO nano-pores, it is demonstrated that the non-repulsive interfacial interactions are the dominant factor in forming the core-shell structure, whereas the unstable three-*T*_g_ scenario originates from other factors, i.e., the deficient extension of the long-range interfacial effect via the weak intermolecular interactions.

**Figure 2:**
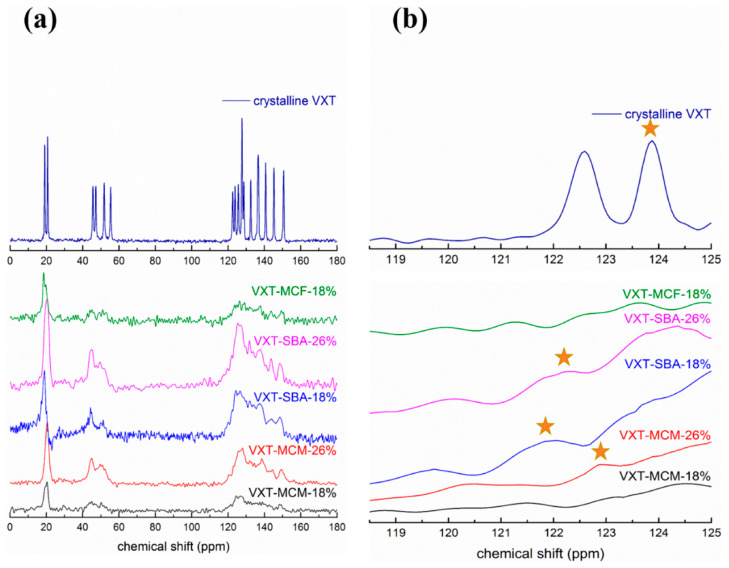
(**a**) ^1^H-^13^C CPMAS NMR results of vortioxetine crystals and vortioxetine-mesoporous silica materials composites; (**b**) chemical shift of the ortho-carbon atom of vortioxetine of vortioxetine in composites. Herein, solid stars represent the NMR signal of the ortho-carbon atom. Adapted from the Ref. [26] with the permission. (Copyright © 2023 American Chemical Society).

Cao et al. investigated the physical stability of amorphous vortioxetine loaded into MCM-41, SBA-15 and mesostructured cellular foam (MCF) [26]. Compared to its pure amorphous form, vortioxetine loaded in these ordered mesoporous silica exhibit enhanced physical stability. These drug-loaded composites exhibit great differences in physical stability, and the order is vortioxetine-SBA-15 > vortioxetine-MCM-41 > vortioxetine-MCF. In addition, the physical stability of confined amorphous vortioxetine is gradually enhanced with the decrease in drug loading. Brunauer–Emmett–Teller (BET) analysis of the ordered mesoporous silica systems with or without vortioxetine-loading reveals that the drug molecules adsorbed in the channels of SBA-15 are in a dispersive state, while they are in an aggregated state in those of MCM-41 and MCF. ^1^H-^13^C solid-state nuclear magnetic resonance (NMR) measurements are performed to investigate the drug–drug interactions. As shown in Figure 2, ^1^H-^13^C NMR spectra of these composites exhibit the broad peaks compared to the vortioxetine crystals. These results are mainly attributed to the carbon nuclei of drug molecules in the pores experience a distribution of environment. In addition, ^13^C chemical shift of the ortho-carbon atom of vortioxetine shows a descending trend in SBA-15, indicating the existence of weaker drug–drug interactions. Consequently, the superior physical stability of amorphous vortioxetine in the channels of SBA-15 can be mainly explained by such weak drug–drug interactions and the dispersive adsorption state.

**Figure 3:**
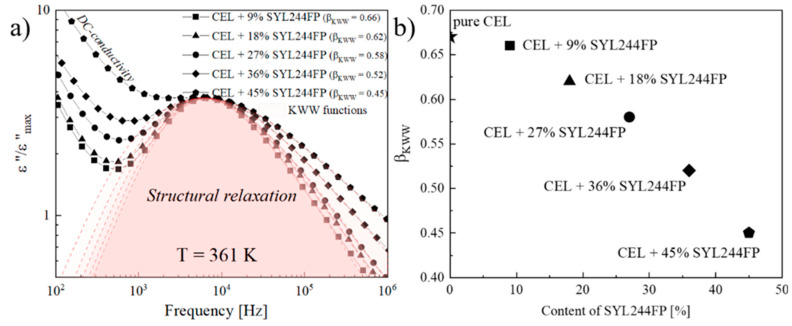
(**a**) The dielectric spectra of celecoxib with different concentrations of Syloid 244FP are recorded at a given temperature. Dashed line represents the shape analysis of the structural relaxation peak in terms of Kohlrausch–Williams–Watts (KWW) function. (**b**) Value of *β*_KWW_ as a function of Syloid 244FP concentration. Adapted from the Ref. [20] with the permission. (Copyright © 2023 Elsevier).

In a very recent study, Kramarczyk et al. explored the effect of mesoporous silica Syloid 244FP on the physical stability and molecular dynamics of amorphous celecoxib [20]. The recrystallization of celecoxib gradually slows down with the increase of mesoporous silica content. Moreover, the fraction of amorphous celecoxib in mesoporous silica will also increase after crystallization. Dielectric analysis reveals that the addition of mesoporous silica does not modify the temperature dependence of the α-relaxation time of celecoxib, but does affect the distribution of α-relaxation peaks. Figure 3 shows the shape analysis of α-relaxation time of celecoxib as a function of Syloid 244FP in terms of Kohlrausch–Williams–Watt (KWW) function. As a measure of the asymmetric distribution of α-relaxation time, the value of *β*_KWW_ decreases with the increase in the content of Syloid 244FP. Meanwhile, the broadening effect of α-relaxation peak of celecoxib indicates an increase in system heterogeneity. Herein, crystallization inhibition of celecoxib in mesoporous silica is proposed to be a result of the formation of a monomolecular drug layer on the silica surface. In addition, it is also proposed that the inhibitory effect of mesoporous silica on crystallization only acts on the drug molecules nearby the silica surface. An extra process addition to α-relaxation process has also been observed in the system containing partially amorphous drugs, which can be identified as the Maxwell–Wagner–Sillars (MWS) polarization in consideration of the inhomogeneity of these systems.

### 2.2. Crystallization under Nano-Confinement

An increasing number of studies have proved that nano-confinement effects can affect the kinetics and thermodynamics of drug crystallization, and can sometimes lead to the formation and stabilization of metastable polymorphs [12,13,27,28,29]. Controlling polymorphic transformation and stabilizing metastable polymorphs via confinement is proposed to provide an opportunity for enhancing solubility and drug delivery. Nanometer-scale pores provide the effect of size constraint during crystallization, resulting in a large ratio of surface area to volume [12]. Compared to the bulk scale counterparts, some properties (melting points, enthalpies of fusion, etc.) of crystals confined in the nano-pores would also be drastically different [12]. The impacts of confinement within nanoscale pores on pathways of crystallization and crystal polymorphism can be particularly prominent, since the dimension of pore is comparable to the critical size of emerging nucleus. The free energy of surface and volume will be in a delicate balance at this tipping point. Consequently, the stability of polymorphs may sometimes be different from that of bulk scale counterparts. Moreover, a snapshot of the earliest stage of crystallization can be obtained by investigating the confinement of crystallization in the nanoscale pores [13]. 

Khimyak and co-workers investigated the solid-state transformation of indomethacin molecules encapsulated in the pores of mesoscopic cellular foam and controlled pore glass [30]. The amorphous indomethacin loaded in these mesoporous systems can be converted into its methanol solvates by adding methanol dropwise and then mixing. Furthermore, stable rare form V will be formed in the mesoscopic cellular foam upon vacuum drying. However, form V of indomethacin encapsulated inside the pores of mesoscopic cellular foam will not be transformed into a more stable form, which is different from the bulk scale counterparts. Nartowski et al. studied the crystallization of flufenamic acid under nano-confinement by using 19^F^ magic-angle-spinning NMR spectroscopy [31]. The confined amorphous flufenamic acid can be observed in MCM-41 (pore size ~3.2 nm) and SBA-15 (pore size ~7.1 nm). For comparison, the form I of crystalline flufenamic acid is found to be formed in the larger pores of mesoscopic cellular foam with a pore size of ~29 nm. More importantly, a molecular liquid-like layer of flufenamic acid exists on the internal surface of these pores, as evidenced by the ^19^F *T*_1_ relaxation times.

As a flexible pharmaceutical molecule, the formation and phase transition of tolbutamide are also investigated by the confined drug molecules in the pores of MCM-41 with a diameter of ~3.2 nm [11]. For the system with low drug loading (≤30 wt%), tolbutamide is stabilized in a highly dynamic amorphous state. With the drug content increasing to 40 and 50 wt%, the highly metastable nano-crystalline from V of tolbutamide can be formed, which is the first report of drug crystallization in such narrow pores. Moreover, the phase transition of tolbutamide from form V to form I^H^ inside the pores of MCM-41 can also be observed by using variable temperature solid-state NMR spectroscopy.

**Figure 4:**
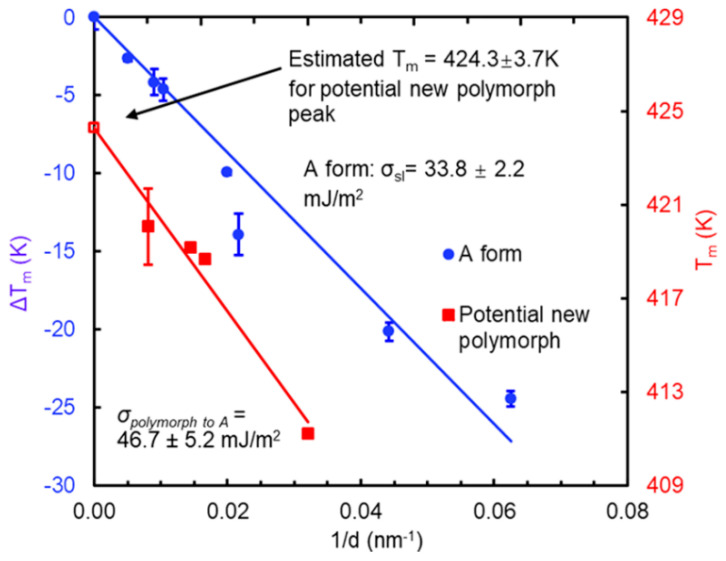
Gibbs–Thomson equation fitting for A and B melting peaks. A and B melting peaks are observed in the melting region for bulk nifedipine and confined nifedipine as a function of pore size. A melting peak is recognized as form A of nifedipine while B melting peak is marked as unknown polymorph transition peak. Adapted from the Ref. [27] with the permission. (Copyright © 2023 American Chemical Society).

Cheng et al. investigated the nano-confinement on the glass transition temperature (*T*_g_) and crystallization of nifedipine by imbibing drug molecules into controlled pore glass [27]. A *T*_g_ depression effect of nifedipine confined in these nanoscale pores can be observed. More than that, nifedipine, which is confined into the smallest pore size (~7.5 nm pore diameter), shows two *T*_g_s, which can be described by a classical two-layer model. The melting point (*T*_m_) of nifedipine polymorphs exhibits a downward trend with an increase of the reciprocal of pore diameter. These depressions of *T*_m_ can be described by the Gibbs–Thomson equation (Figure 4). Upon heating, two *T*_m_ endothermal peaks of nifedipine are observed in the mesoporous systems, showing a pore size greater than 50 nm. The analysis of *T*_m_ by the Gibbs–Thomson equation indicates that the *T*_m_ of the second peak, represented as the blue rounds in Figure 4, is attributed to the form A of nifedipine. The lower peak, represented as red squares in Figure 4, is mainly attributed to the transformation of an unknown polymorph into form A under nano-confinement of 50–198 nm size [27]. 

Recent studies have also revealed that nano-confinement can also affect the pathways of polymorphic phase transformation [28,32,33]. For instance, form III of acetaminophen, an unstable form in bulk, can be stabilized in the nano-pores of the controlled pore glasses [34]. Moreover, the solid–solid polymorphic transformation from form III to form II is also effectively inhibited in these nano-pores, even during the heating process. Hamilton et al. reported that the pore size can affect the stability of confine *β*-form of glycine [35]. This metastable form exhibits a slow polymorphic transformation rate to its α-form in the 55 nm pores. Furthermore, *β*-form of glycine is also found to be indefinitely stable in the nano-pores smaller than 30 nm in size. In addition, Zhang et al. reported that the extremely unstable form VIII of flufenamic acid can be favorably formed under the nanoscale confinement [32]. They also found that the polymorphic phase transformation pathways of flufenamic acid strongly depend on the pore size of the controlled pore glass used [32]. A polymorphic transformation pathway from form VIII to form II and then to form I was observed in the nanocrystals embedded in the controlled pore glass with 30–50 nm diameter pores. For larger pore size (100–200 nm pore diameter), a proceeding of polymorph transformation from form VIII → form IV → form III can be observed. Moreover, it was found that form VIII is directly converted into form III in the bulk phase. 

In a very recent study, Cao et al. reported the preparation of pure metastable form α of vortioxetine hydrobromide in silica nano-pores [36]. By using the specific solvent vapor, amorphous vortioxetine hydrobromide is crystallized into its crystal form α. The polymorphic outcomes can be strongly affected by the properties of solvent used and the size of silica pores. For silica pores not larger than 50 nm, solvent vapors with a larger molecular volume facilitate the formation of form α. However, in the pores with larger size, the polymorphic behavior of vortioxetine hydrobromide is dominated by the capacity of solvent hydrogen donor. A solvent showing a low hydrogen bond capacity is conductive to the formation of form α. The confined form α exhibits an enhanced physical stability in comparison with the bulk form under various test temperature–relative humidity conditions. Encouragingly, the metastable α form of vortioxetine hydrobromide can be effectively maintained for at least one month. Moreover, α form of vortioxetine hydrobromide under nano-confinement shows a faster dissolution rate compared to its bulk form. 

Fellah et al. compared the crystallization of 21 highly polymorphic compounds from bulk and confined melts [28], among which 19 polymorphic systems confined into the nano-pores from 8 to 100 nm pore size are the polymorphs that dominate crystallization from the bulk melt at similar temperature. The amount of polymorphs formed in these nanoscale pores is less than that formed from bulk melt. However, some new polymorphs, which cannot be detected by other crystallization methods, can be generated from the melt under nano-confinement. Recent study also demonstrates that the racemic praziquantel-glutaric acid cocrystal can be formed in the nano-pores of SBA-15 with a pore size of ~5.6 nm [37]. Solid-state NMR reveals that the phase of praziquantel-glutaric acid cocrystal exhibits higher mobility, while the IR spectra show that it still retains the cocrystal-like features. 

## 3. In Vitro and In Vivo Performance of Mesoporous Drug Delivery Systems

A drug loaded in mesoporous silica can sometimes exhibit enhanced solubility and dissolution, which makes it a promising formulation strategy for poorly water-soluble drugs [4,38,39]. For instance, drug molecules can exist in amorphous form in the nanoscale pores or on the silica surface of mesoporous materials. A faster dissolution rate is expected because of the higher energy and lack of crystal lattices of amorphous drugs. Taking asarone, a hydrophobic drug, as an example, after it is loaded into SBA-15 by supercritical carbon dioxide technique, the in vitro dissolution and in vivo bioavailability are significantly improved compared with its micronized crystalline counterparts [40]. 

Dissolution from mesoporous drug delivery system is a very complex process, which can be affected by various factors, including mesoporous structure, pore size, surface functionalization, drug-mesoporous surface interaction, etc. McCarthy et al. investigated the effects of the porous architecture of mesoporous SBA-15 and non-porous Aerosil^®^200 on the adsorption and dissolution processes [41]. Compared to the surface of mesoporous materials, non-porous surface bounds a larger amount of drugs. For non-porous silica, the adsorption isotherms are useful for understanding the drug adsorption and release behaviors. However, the quantity of drug remaining on the surface of mesoporous silica after dissolution is much higher than the predicted value using adsorption isotherm data. These results are proposed to be mainly attributed to the parts of drug molecules tightly bound to the surface of silica or attached to the sites those are inaccessible for the dissolution media. In a recent study, the release behaviors of atenolol from microporous zeolites faujasite (FAU), zeolite beta (BEA) and mesoporous MCM-41 were investigated from both the experimental and theoretical perspectives [42]. Atenolol is released from FAU in phosphate buffer in zero-order for 24 h, and sustained release can still be observed at least in the next 48 h. Herein, all atenolol–zeolite combinations are found to exhibit prolonged drug release behaviors. Molecular dynamics simulation has been demonstrated to be a potential screening tool for predicting the effect of steric hindrance in these atenolol–zeolite combinations and identifying the suitable frameworks for prolonging drug release. 

Garcia-Bennett et al. investigate the interactions between silica surface and loaded amorphous drug by combining structural and calorimetric characterization with atomic pair distribution function analysis [43], in which, atomic pair distribution function analysis is used to identify the local ordering of drug molecules. The release kinetics can be rationalized by several drug factors including crystallization properties, molecular size, and *T*_g_. Additionally, the filling degree of meso- and micro-pores is also an important factor influencing the drug release process. Wang and co-workers explored the feasibility of uniform mesoporous carbon spheres (UMCS) and fibrous ordered mesoporous carbon (FOMC) as carriers of lovastatin, a poorly water-soluble drug [44]. In their study, UMCS shows a three-dimensional pore system, while FOM has a two-dimensional hexagonal mesoporous structure, both of which exhibit high drug loading. Compared to the pure crystalline form, lovastatin loaded in UMCS and FOMC shows a faster dissolution rate. Moreover, it is shown that the mesoporous carrier with larger pore size contributes to faster dissolution rate. 

Besides the porous architecture, drug loading is also one of the key factors affecting the dissolution behavior of drugs from the mesoporous carriers. Hate et al. prepared atazanavir-loaded SBA-15 systems with various drug contents by incipient impregnation [45]. Herein, atazanavir is identified as amorphous form and can form intermolecular interactions between its carbonyl group and the silanol group of SBA-15. The drug release from SBA-15 exhibits incomplete release in the closed compartment dissolution test, largely originated from the drug adsorption tendency. In contrast, in the coupled dissolution-adsorption test, atazanavir in this mesoporous system exhibits a complete release over a 240 min time period, which is mainly attributed to the diffusion of drug across the membrane. In addition, the higher solubility of atazanavir at lower pH also leads to further improvement of drug release when the formulation is firstly added to the gastric pH condition of a fasting state followed by pH shifting to intestinal condition. However, a poorer overall absorption behavior can be observed in the atazanavir-loaded mesoporous silica systems in comparison with polymer-based ASD formulations.

**Figure 5:**
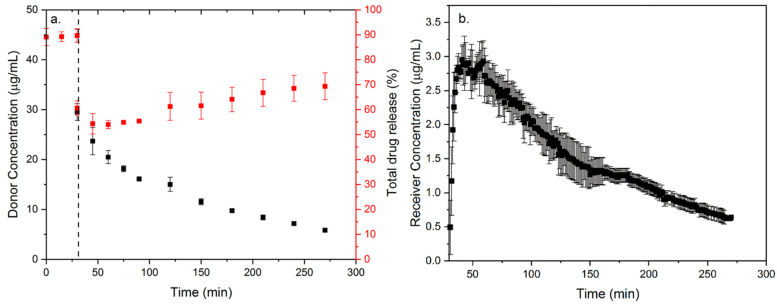
(**a**) Donor, total drug release and (**b**) receiver concentration of ketoconazole mesoporous silica-based formulations during dissolution-absorption measurements with pH shifting from pH 1 to 6.8 after 30 min. Donor concentration exhibits an abrupt decline and is followed by a further gradual decline. Receiver concentration exhibits a maximum and a gradual decline in concentration follows. Only slight increase of total drug release (by 10%) can be observed over 4 h. Adapted from the Ref. [46] with the permission. (Copyright © 2023 American Chemical Society).

Incomplete drug release is a widely reported phenomenon in mesoporous drug delivery systems, and is also proposed to be strongly related to the drug–silica surface interactions [46]. Herein, the strength of molecular interactions between drug molecules and silica surface mainly depends on several factors, including silica surface chemistry, pH of the dissolution medium, drug chemistry, and ionization state. Hate et al. investigate the effects of electrostatic interaction between drug molecules and silica surface on the dissolution of weakly basic drugs from mesoporous silica-based formulations as a function of medium pH [46]. Compared to the formation of hydrogen bonding interaction, drug–silica surface electrostatic interaction will lead to higher adsorption and lower drug release. Meanwhile, the increase of pH value in the dissolution medium results in the increase of negative charge on silica surface and the adsorption of positively charged drugs. In the case of atazanavir mesoporous silica-based formulations, an abrupt decline and a followed further gradual decline can be observed in donor concentration upon changing the pH from 1 to 6.8 (Figure 5a). These phenomena are mainly attributed to the simultaneous adsorption of drugs across the membrane. Differently, the receiver concentration reaches a maximum value and then gradually decreases, reciprocating the decrease in donor concentration (Figure 5b). From the perspective of total drug release, only a slight increase (10%) is observed over the 4h adsorption measurement. This slight increase suggests that only a small amount of additional drug release is mainly due to simultaneous absorption. In addition, it is also proposed that the formation of electrostatic interactions will diminish the sink condition provided by the adsorptive environment. 

**Figure 6:**
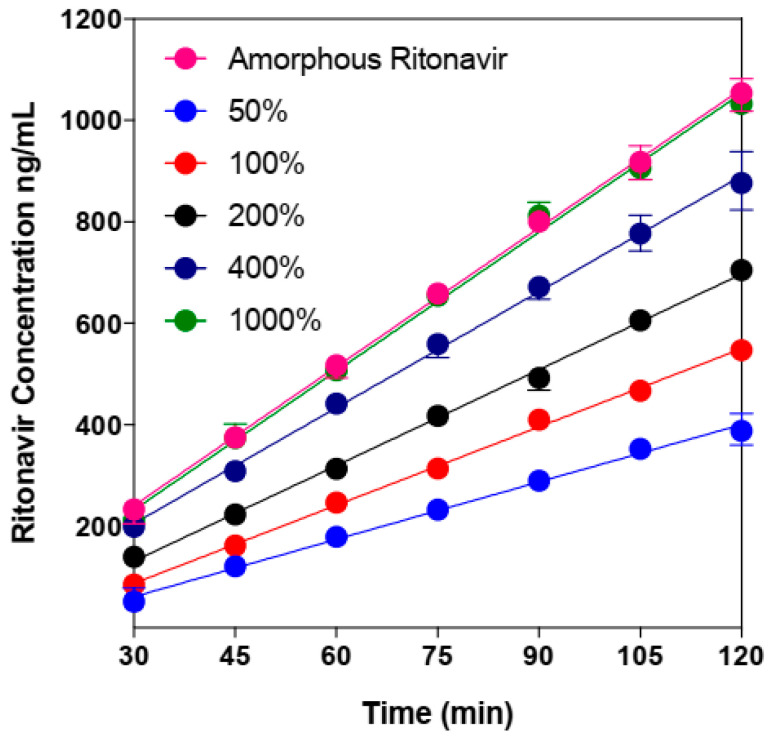
Ritonavir concentration versus time profiles in the receiver compartment in vitro diffusion studies for the ritonavir-loaded SBA-15 systems by using a side-by-side diffusion cell with different drug doses (% of its amorphous solubility). A known quantity of sample is first added into the donor compartment to obtain the desired total drug concentration. Then, drug concentration in receiver compartment is monitored as a function of time through periodically withdrawing 100 µL aliquots and diluting with 50 µL acetonitrile. Adapted from the Ref. [48] with the permission. (Copyright © 2023 American Chemical Society).

The supersaturation potential of drug in the mesoporous drug delivery systems has also attracted considerable research interests due to its great implications on bioavailability [47,48]. For instance, Zhang et al. report the enhanced dissolution and supersaturation of drugs in the drug-loaded mesoporous magnesium carbonate systems [47]. Among them, the areas under the dissolution concentration–time curve of celecoxib, cinnarizine and griseofulvin increase by twenty-five times, five times, and two times, respectively, in comparison with those of their crystalline form. Dening et al. systemically investigated the release behavior and supersaturation of ritonavir from mesoporous SBA-15 silica particles by undertaking in vitro dissolution and membrane flux studies of samples [48]. The supersaturated solution of ritonavir can be generated with the release from the mesoporous SBA-15 particles, while its drug release is always incomplete, even under the sink condition. Moreover, with the increase in theoretical supersaturation ratio and drug dose of these SBA-15 formulations, the percentage of drug release decreases significantly (Figure 6). An equilibrium is proposed to be existed between drugs in the solution and adsorbed to the silica surface. McCarthy et al. investigated the mechanism of drug–silica interactions and the adsorption behaviors in a supersaturated solution of indomethacin and its methyl ester with a similar drug molecular structure but different capability of hydrogen bonding [49]. It is found that hydrogen-bonding interactions can be formed between the acceptor carbonyl groups of the drug and the donor groups on the silica surface. However, in the presence of the hydrogen bond donor of a drug, its adsorption on the silica surface cannot be enhanced. In addition, there is no evidence to support the view that drugs can be adsorbed on a silica surface via non-specific hydrophobic interactions. The equilibrium between drugs existing in a solution and adsorbed on a silica surface is proposed to be strongly related to the activity of drugs in a solution. Under supersaturated conditions, the high tendency of drug adsorption on a silica surface, in turn, limits the extent of drug release.

The inclusion of a third component to generate ternary mesoporous systems may modify the performance in vitro and in vivo [41,50,51,52]. For instance, in the presence of sodium dodecyl sulphate, a surfactant, the release of sulphamethazine from a silica surface can be enhanced [41]. This phenomenon is considered to be a result of wetting characteristics of the media and the adsorption of surfactant on the silica surface. Le et al. investigated the effects of drug overloading within mesoporous silica on drug release and thermal properties [51]. Drug overloading can effectively increase the maximum dissolution attained. In addition, incomplete drug release can also be observed, even at low drug loading, which is mainly attributed to the reversible adsorption to the surface of mesoporous silica. The presence of HPMCAS, a polymeric precipitation inhibitor, cannot promote the amorphization of drugs in mesoporous silica. Moreover, partial coating of HPMCAS on the exterior surface of mesoporous silica particles will lead to slower drug release. In the ternary system containing glibenclamide, HPMCAS, and mesoporous silica, the extent and duration of drug supersaturation have also been similarly improved [52]. One of the main attributes of enhanced dissolution performance is the formation of strong drug–polymer interactions. The premature release and precipitation of glibenclamide can also be effectively prevented by the immobilization of drug-loaded silica on HPMCAS plates. Moreover, the proximity of drug–polymer at the disintegration of this ternary system facilitates the rapid onset of precipitation inhibition.

Munir et al. prepared the binary and ternary solid dispersions of flurbiprofen using unordered mesoporous silica and gelucire, aiming to improve drug solubility and prevent the ulcerogenic effect [53]. Among these prepared samples, in the presence or absence of 25% *w*/*w* gelucire, the formulations with a drug–silica ratio of 1:1 successfully incorporate drug molecules into ASD without any obvious interactions. In these systems, flurbiprofen exhibits increased solubility and almost complete release within 30–45 min. Herein, the ternary systems containing 25% gelucire are observed to exhibit the reduction in gastric lesion index, indicating the superior gastro-protective effects in vivo. Additionally, the gastro-protective effects of these ternary systems can also be evidenced by the normal epithelial cells and partially protected mucosa shown in the histological images of stomach lining.

**Figure 7:**
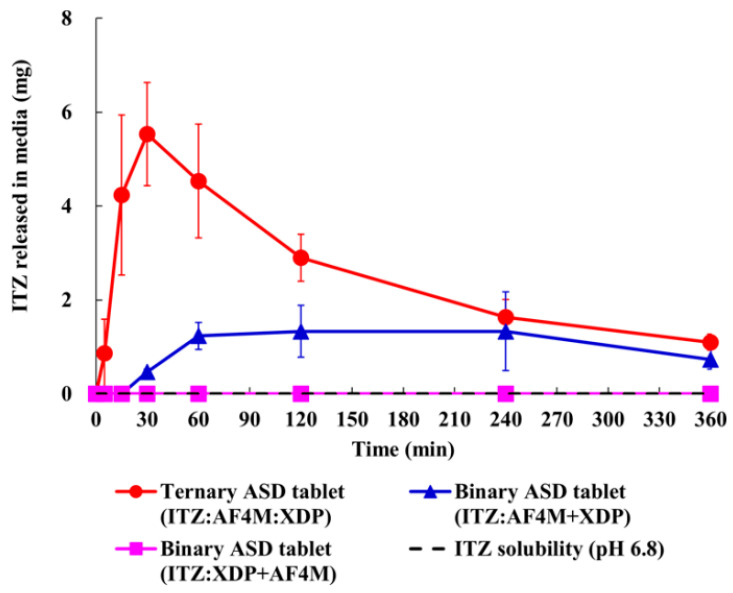
Dissolution behaviors of itraconazole binary and ternary ASD tablets by using USP apparatus II under non-sink conditions. Before testing, 500 mL of pH 6.8 phosphate buffer is added to the dissolution vessel and maintained at 37 °C. Paddle speed is set to 100 rpm. Samples containing 100 mg itraconazole first pass through 212 μm sieves and are immediately added into the vessel. Aliquots are collected at time points of 15 min, 30 min, 1 h, 2 h, 4 h, and 6 h. Adapted from the Ref. [54] with the permission. (Copyright © 2023 American Chemical Society).

Ternary amorphous solid dispersions (ASDs) containing itraconazole (ITZ), high-viscosity hypromellose (HPMC), and mesoporous silica can also be prepared by hot melt extrusions [54]. The superior ability in maintaining the supersaturation of itraconazole can be observed in these ternary systems. Tablets showing a patient-friendly size are designed to contain a high content of these ternary systems with both sufficient tablet hardness and rapid disintegration. Similar to the binary ASD granules, the drug dissolution from the tablets also exhibits a high maintenance ability of drug supersaturation. Moreover, ternary ASD tablets exhibit immediate and higher drug release in comparison with binary ASD tablets (Figure 7), which is mainly attributed to the differences in particle size of drug/high-viscosity hypromellose. Itraconazole and high-viscosity hypromellose can easily form nano-sized particles by hot melt extrusion into/onto mesoporous pores during the adsorption process, which contributes to the immediate drug release from the ternary ASD tablets.

It is also reported that the addition of mesoporous silica facilitates the generation of ternary ASDs by using hot melt extrusion [55], which depends on the grade employed and the bulk concentration of mesoporous silica. The spreading extent of drug–polymer mixtures to particles is limited by the bulk density and pore size of mesoporous silica grades along with the dynamic viscosity of mesoporous silica and Soluplus. Compared to its crystalline counterparts, ternary ASD formulations containing 25 wt and 50 wt% of felodipine exhibit the enhanced drug stability and solubility. A two-step or three-step release pattern can also be observed in the ternary systems. On the basis of the Higuchi model, these release patterns are mainly attributed to the drug release from the polymer matrix and external silica surface followed by drug release from the silica pore structure. Herein, the mechanism of drug release is proposed to be governed by a complex quasi-Fickian release mechanism, in which, multiple drug release mechanisms are occurring concurrently and consequently. 

Xi et al. prepare indomethacin solid dispersion by using two different polymers, hydroxypropyl methylcellulose (HPMC) and Kollicoat IR in combination with mesoporous silica nanoparticles [56]. Herein, indomethacin loaded into these designed carriers is characterized as amorphous form. Compared with pure crystalline drug, the in vitro dissolution rate of indomethacin in these designed carriers exhibits a three-fold increase. Moreover, HPMC and Kollicoat IR also play a prominent role in improving the gastrointestinal absorption efficiency and bioavailability of indomethacin in these solid dispersions containing mesoporous silica nanoparticles. Mitran et al. reported that the release kinetics of water-soluble therapeutic agents from mesoporous silica can be tailored by adding hydrophobic excipient 1-tetradecanol [57]. Herein, the addition of tetradecanol is found to slow down the drug release, which is strongly related to the presence of liquid fatty alcohol interfacial layer. This effect is proposed to be independent of the pore arrangement of drug carrier, and is noticed in both hexagonal MCM-41 and cubic KIT-5 mesoporous silica. Moreover, no significant toxicity can be observed for the tetradecanol-containing mesoporous silica.

In addition to polymer-based ASD, mesoporous silica has also been reported to affect the dissolution of coamorphous and co-crystal systems [37,58]. For instance, compared to pure co-crystal form, the sustained solubilization of praziquantel SBA-15/praziquantel-glutaric acid cocrystal composites can be observed in the presence of cellulosic polymer [37]. Budiman et al. prepared a ternary formulation in which the ritonavir–saccharin coamorphous system is incorporated into mesoporous silica through solvent evaporation [58]. Ritonavir and saccharin are demonstrated to be monomolecularly incorporated into mesopores, which is evidenced by the absence of *T*_g_ of this coamorphous system on the DSC curve. ^13^C solid-state NMR spectra reveal the formation of hydrogen bonding interactions between thiazole nitrogen of ritonavir and amine proton of saccharin. Moreover, co-incorporation of saccharin into the pores of mesoporous silica reduces the local mobility of thiazole group of ritonavir via the formation of hydrogen bonding interactions. The ritonavir-saccharin coamorphous system at a molar ratio of 1:1 effectively maintains its amorphous form for 30 days of storage in mesoporous silica, even in the presence of high temperature and humidity. In addition, these coamorphous systems in mesoporous silica can maintain the supersaturation of ritonavir for a longer time compared to the pure amorphous ritonavir loaded in mesoporous silica.

USP Type II (paddle) dissolution apparatus is widely used to investigate the in vitro dissolution of mesoporous silica system, while the ability to forecast the bioavailability in vivo still remains in-depth investigations. McCarthy et al. examine the ability of three different in vitro dissolution apparatuses to predict the oral bioavailability of fenofibrate-loaded mesoporous silica systems in vivo [59]. Herein, it is found that dissolution apparatus and experimental design can strongly affect the in vitro performance of the mesoporous formulations. In vitro/in vivo relationship analysis shows that the USP IV transfer model, incorporating simulated gastric fluid (SGF) into FaSSIF-V2 medium transfer is the best predictor of bioavailability in comparison with paddle apparatus and flow-through cell apparatus. This is mainly attributed to its special hydrodynamic properties and its ability to better simulate gastrointestinal transit.

Several studies also investigate the biodistribution, clearance, and biocompatibility of mesoporous materials in vivo [60,61,62,63]. Huang et al. found that intravenously administrated mesoporous silica nanoparticles (MSNs) are mainly enriched in the liver, spleen, and lung [60]. Particle shape is found to strongly affect the in vivo behaviors of these MSNs. Short-rod MSNs are easily enriched in the liver, while the long-rod form is mainly concentrated in the spleen. MSNs are mainly excreted by urine and feces, and the short-rod MSNs exhibit a more rapid clearance rate in comparison with the long-rod form. In a very recent study, Moya and coworkers prepared MSNs with both the core and surface labelling [61]. MSNs can accumulate in the liver and spleen. The degradation products and silicate bearing the radioisotope can be found in the bones and probably in the lungs.

## 4. Modification and Design of Mesoporous Systems and Its Implication for Drug Release Control

It is well accepted that mesoporous silica nanoparticles can be developed as promising targeted nanomaterials for cancer theranostics [64,65,66]. Their advantages in such applications are mainly attributed to various morphologies, large surface areas, pore diameters and arrangement, and different surface functionalizations. Several excellent reviews summarize the recent advances in the functionalization of mesoporous silica nanoparticles by small molecules, polymers, proteins, aptamers, saccharides, peptides, antibodies, etc. [64,67,68,69]. The molecules loaded or entrapped in these surface-functionalized mesoporous silica nanoparticles are expected to exhibit the on-desire release behavior. Various surface functionalizations of mesoporous silica nanoparticles lead to the seemingly endless prospects of advanced nano-constructs in cancer targeting, imaging, and therapy [64,67,68,70]. Herein, this part of the present review focuses on the effects of surface functionalization on the release behaviors of small molecule drugs in recent studies.

Vorinostat, a drug mainly used for the treatment of cutaneous T-cell lymphoma, is encapsulated within mesoporous silica nanoparticles, which are modified with different functional groups [67]. The solubility, permeability and in vitro anti-cancer efficacy of vorinostat in mesoporous silica nanoparticles (MSNs) are assessed [71]. A 2.6-fold increase in the solubility of vorinostat encapsulated in the pristine mesoporous silica nanoparticles can be observed in comparison with the free drug form. The solubility of vorinostat can be further increased if these mesoporous silica nanoparticles are modified by silanes with amino or phosphonate terminal functional groups. The mesoporous silica nanoparticles-based formulations are also found to significantly enhance the permeability of vorinostat, particularly for amino functional group-modified mesoporous silica nanoparticles (~four-fold increase). Moreover, compared with the free drug, amino-modified MSNs exhibit improved anti-tumor activity in both colorectal and cutaneous T cell lymphoma cells.

Lee et al. reported the high drug loading (up to 97%) and precisely controlled drug release of doxorubicin in mesoporous silica nanoparticles modified by amine moieties, gold nanoparticles and albumin [72]. In situ Raman spectroscopy and mapping were used for monitoring the drug release and visualizing the distribution of targeted drugs in cells. Goscianska et al. investigated the adsorption and release behaviors of ibuprofen from lanthanum-modified ordered mesoporous silica SBA-15 and KIT-6 [73]. Herein, lanthanum strongly determines the structural and textural properties of silica. With the increase of average pore diameter and percentage content of lanthanum, the storage capacity of modified silica increases. The high coverage of lanthanum on the surface of mesoporous materials can lead to an increase in the loading amount and release rate of ibuprofen. 

A novel mesocellular carbon matrix (MSU-FC) with large pore size and 3D structure is prepared to deliver the poorly water-soluble drugs [74]. Celecoxib, incorporated into MSU-FC via solvent immersion/evaporation method, shows an approximately 9-fold increase in water solubility compared to its pure crystalline form. Compared with crystalline celecoxib and celecoxib-loaded conventional mesoporous carbon particles, celecoxib loaded in MSU-FC exhibits accelerated immediate release. Moreover, higher bioavailability and lower toxicity can also be observed in celecoxib-loaded MSU-FC systems. Han et al. prepared a novel type of mesoporous silica nanoparticles, which exhibits the core-shell structure (CSMSNs) [75]. Celecoxib loaded in CSMSNs not only shows improved dissolution and enhanced bioavailability by changing its needle-like crystal form, but also has good biocompatibility, which can be corroborated in the gastric mucosa irritation study. Celecoxib crystals can be controlled by the nano-channels of CSMSNs, which facilitates better cumulative drug dissolution (86.2% release in SIF solution within 60 min). Moreover, celecoxib loaded in CSMSNs exhibits a 9.9-fold increase in AUC in comparison with that of pure drug, and a 1.89-fold increase in comparison with its commercial preparation.

## 5. Conclusions and Future Outlook

**Figure 8:**
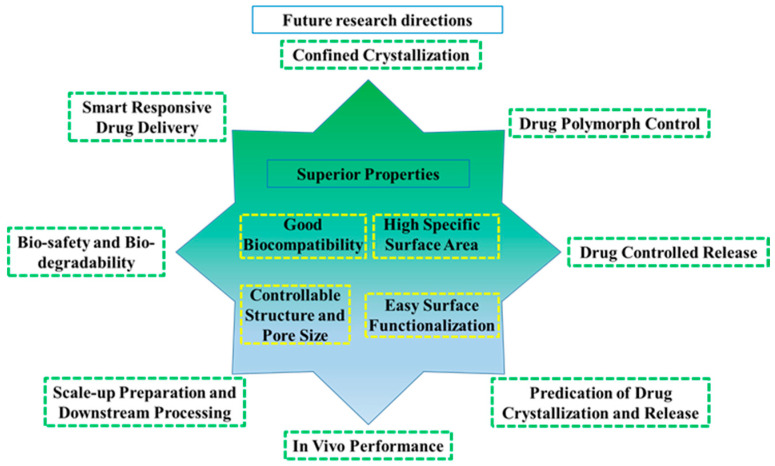
Overview of superior properties and possible future research directions of the mesoporous materials in the pharmaceutical field.

In conclusion, this review provides detailed descriptions and examples regarding the role of mesoporous materials in affecting the molecular dynamics, crystallization, in vitro performance, and in vivo performance of amorphous and/or crystalline drugs. Table 1 summary the API and mesoporous materials discussed in present work. Although considerable efforts have been made in the field of mesoporous drug delivery systems, several challenges still need to be addressed to develop robust mesoporous formulations. Figure 8 summarizes the superior properties of mesoporous materials and the possible future research directions in the field of pharmaceutical science. One of the most important issues is to investigate the thermodynamic and kinetic factors of confined crystallization in mesoporous systems in depth. In order to better understand the role of the dimensional and interfacial factors of mesoporous materials in the control and discovery of polymorphs, more systematic studies are also urgently demanded. Rational design of mesoporous structure and surface functionalization is also required o obtain the desired pharmaceutical properties.

Future work is warranted to better understand the release of drugs from mesoporous materials, whether sustained or rapid release. The adsorption/desorption behaviors of drugs under various supersaturations should be qualitatively and quantitatively evaluated. In addition, the impacts of various physicochemical properties of different drugs, as well as the molecular interactions between drugs and mesoporous carriers, on the adsorption/desorption behaviors of drugs should be further investigated. More complete and clearer pictures of drug adsorption/desorption in mesoporous carriers need to be established. Moreover, more sophisticated models are also required for predicting the release of drugs from mesoporous systems, particularly for biologically relevant media. 

The safety and efficacy of mesoporous materials should be taken into deep consideration to realize the commercial feasibility of mesoporous formulations. A large number of in vivo studies are still urgently required to study the potential role of mesoporous materials in biological environments. Bio-safety and bio-degradable mesoporous materials also require considerable investigation and development. Furthermore, the scale-up preparation and downstream processing of mesoporous formulations into commercial forms is still in need of exploration. Technologies utilized for producing mesoporous formulation at industrial scale require further development as well. With the in-depth understanding and further development of mesoporous drug delivery systems, it is well expected that such mesoporous formulations will ultimately be translated into the drug market.

## Figures and Tables

**Table 1:** API and mesoporous materials discussed in the present study.

API	Mesoporous Materials	Reference
Indomethacin Griseofulvin	anodic aluminum oxide (AAO)	[10]
Tolbutamide	MCM-41	[11]
Celecoxib	Syloid 244 FP (SYL244FP)	[20]
Ezetimibe	Aeroperl 300 Neusilin US2	[21]
Simvastatin	Syloid XDP 3050 Syloid 244 FP	[22]
Mangiferin	Syloid^®^ XDP 3050	[23]
Menthol	MCM-41 SBA-15	[24]
Haloperidol Carbamazepine Benzamide	Parteck SLC 500 Neusilin US2 Syloid^®^ XDP 3050 Aeroperl 300	[25]
Vortioxetine	MCM-41 SBA-15 mesostructured cellular foam (MCF)	[26]
Flufenamic acid	controlled pore glass (CPG)	[32]
Nifedipine	controlled pore glass (CPG)	[27]
Oxalyl dihydrazide Nicotinamide Isonicotinamide Coumarin Paracetamol Acridine Theophylline Carbamazepine Dantron Sulfapyridine Imidacloprid ROY Tolfenamic acid Tolbutamide Chlorpropamide Flufenamic acid Galunisertib Aripiprazole Sulfathiazole Sulfameter	controlled pore glass (CPG)	[28]
[1-^13^C] glycine	SBA-15 with wall-embedded TEMPO radicals	[29]
Indomethacin	mesoscopic cellular foam (MCF) controlled pore glass (CPG)	[30]
Flufenamic acid	MCM-41 SBA-15 mesoscopic cellular foam (MCF)	[31]
Acetaminophen	controlled pore glass (CPG)	[34]
Glycine	controlled pore glass(CPG) porous polystyrene-poly(dimethyl acrylamide) (p-PS-PDMA) monoliths	[35]
Vortioxetine Hydrobromide	MCM-41 Porous silica particles	[36]
Praziquantel Praziquantel-glutaric acid cocrystal	SBA-15	[37]
Silymarin	mesoporous silica nanospheres with a 3D dendritic pore structure	[38]
Ibuprofen	mesoporous silica particles	[39]
Asarone	SBA-15	[40]
Sulphamethazine	SBA-15 Aerosil^®^200 (non-porous)	[41]
Atenolol	MCM-41 microporous zeolites FAU and BEA	[42]
Albendazole Hydrocortisone Indomethacin	MCM-41 MCM-48 SBA-15	[43]
Lovastatin	uniform mesoporous carbon spheres (UMCS) with 3D pore system fibrous ordered mesoporous carbon (FOMC) with two-dimensional hexagonal mesoporous structure	[44]
Atazanavir	SBA-15	[45]
Ketoconazole Clozapine Atazanavir	SBA-15	[46]
Celecoxib Cinnarizine Griseofulvin	mesoporous magnesium carbonate (MMC)	[47]
Ritonavir Lopinavir	SBA-15	[48]
Indomethacin and Indomethacin methyl ester	SBA-15	[49]
Itraconazole	SBA-15	[50]
Felodipine Furosemide	Syloid XDP 3050	[51]
Glibenclamide	mesoporous silica	[52]
Flurbiprofen	Syloid 244 FP Syloid AL1 FP	[53]
Itraconazole	Syloid XDP 3050	[54]
Felodipine	Syloid 244 FP Syloid AL1 FP Syloid XDP 3050	[55]
Indomethacin	mesoporous silica nanoparticles	[56]
Metoprolol	MCM-41	[57]
Ritonavir -saccharin coamorphous system	Taiyo’s mesoporous silica (TMPS)	[58]
Vorinostat	MCM-41 ordered mesoporous silica nanoparticles	[71]
Ibuprofen	Cubic mesoporous silica KIT-6 SBA-15	[73]
Celecoxib	Three-dimensional large-pore mesoporous carbon matrix	[74]
Celecoxib	Core-shell mesoporous silica	[75]
